# Association of advanced age and cancer history with autoimmune disease in melanoma patients: a cross-sectional study

**DOI:** 10.1186/s12885-021-09001-1

**Published:** 2021-12-06

**Authors:** Aaron N. Holmes, Helen Swede, Wendy M. Feer, Donna Comins Pike, Xiaoyan Wang, Upendra P. Hegde

**Affiliations:** 1 University of Connecticut Health Center, 263 Farmington Avenue, Farmington, CT 06030 USA; 2 Sema4, Mount Sinai Health System, Stamford, CT USA

**Keywords:** Melanoma, Autoimmunity, Immunotherapy, Aging, Immune checkpoint inhibitors

## Abstract

**Background:**

Immune-related adverse events (irAEs) are a major toxicity of immune checkpoint inhibitors. Studies have reported that pre-existing autoimmunity increases the risk of irAEs, but it remains unknown which clinical factors are linked to auto-immune disorders in cancer patients. This study aimed to evaluate if the prevalence of autoimmune diseases varied by specific cancer history and advanced age.

**Methods:**

Our cross-sectional medical record review consisted of 291,333 patients (age, ≥18 years) treated between 2000 and 2018. Patients were classified into four study groups (melanoma only, non-cutaneous solid cancer only, melanoma and non-cutaneous cancer, and no cancer history). Dependent variable was the presence of ≥1 autoimmune disorders based on 98 conditions using 317 ICD codes.

**Results:**

Non-cutaneous cancer, in the absence or presence of melanoma, was associated with a higher prevalence of autoimmunity (16.5, 95% CI 16.1–16.9; 20.0, 95% CI 18.3–21.7, respectively) compared to the rates in patients with melanoma only and those without cancer history (9.3, 95% CI 8.6–10.0; 6.2, 95% CI 6.1–6.3, respectively). Among patients with metastases at initial presentation, those in the melanoma and non-cutaneous cancer group had a prevalence of 24.0% (95% CI 20.1–27.9) compared to 19.1% (95% CI 17.2–21.0) in those without metastases. Multiple logistic regression demonstrated that patients > 75 years exhibited the highest odds of autoimmunity relative to other age groups, with age 18–34 as the referent (OR, 1.78, 95% CI 1.67–1.89).

**Conclusions:**

Among patients with melanoma, the greatest prevalence of autoimmunity occurred with advanced age and a history of non-cutaneous cancer.

**Supplementary Information:**

The online version contains supplementary material available at 10.1186/s12885-021-09001-1.

## Background

Clinically effective anti-cancer immune therapy has become possible with the advent of immune checkpoint inhibitors (ICIs). Such therapies have improved outcomes in melanoma, renal cell carcinoma, non-small cell lung cancer, and other cancers [1]. However, patients on this therapy are at risk of developing immune-related adverse events (irAEs) that at times can be severe, unpredictable, and life threatening. The fatality rates of irAEs for anti-CTLA-4 and anti-PD-1 therapy are 1.08 and 0.36%, respectively [2].

Meta-analyses have established that pre-existing autoimmunity increases the risk of irAEs following ICI therapy [3, 4]. Since advanced age predisposes to both melanoma and autoimmunity, there are concerns of enhanced autoimmunity in senior patients with melanoma and other cancers receiving ICIs [5–7]. Interestingly, meta-analyses of large clinical trials do not see differences in irAEs by age [8–10]. By contrast, retrospective studies and meta-analyses of case reports from “real-world” experiences are mixed with some reporting higher autoimmunity in senior patients [10–12]. Some significant limitations exist in past work, as clinical trials highly select patients and many real-word case reports are low-power studies due to low sample sizes. Large retrospective studies of health centers in the community may aid in resolving this controversy.

An understanding of underlying autoimmunity by age and cancer diagnosis may help establish irAE risk in different patient populations. To this end, we examined prevalence of autoimmunity by age and cancer diagnosis from a large single-institution database. We decided to focus on melanoma as our model cancer, because melanoma is an immunogenic cancer that responds well to ICIs, and its incidence has sharply increased in recent years among senior patients.

## Methods

### Study design

We conducted a cross-sectional study of 291,333 patients aged 18–106 years old who were treated at in-patient or out-patient sites of the University of Connecticut Health Center between 2000 and 2018. Patient information was abstracted from a GE Centricity IDX database of the electronic medical record. The sample consisted of 36,219 cancer patients and a random sample of 255,114 patients without cancer history.

### Independent variables and covariables

Patients were organized into four cancer-related study groups, those with: (1) primary melanoma only (*n* = 6543), (2) non-cutaneous malignant neoplasms only (*n* = 27,630), (3) both melanoma and non-cutaneous malignant neoplasms (*n* = 2046), and (4) patients without a cancer history (*n* = 255,114). International Classifications of Diseases, Ninth and Tenth Revisions (ICD-9, ICD-10) codes were used as follows: melanoma (ICD-9172.x; ICD-10 D03.xx, C43.x) and non-cutaneous neoplasms (ICD-9140–239 aside from 172 to 173.x, 196-198.x, 216.x. 232.x, 238–239.x; ICD-10 C00-D48 aside from C43-44.x, C77-C79.x, D03-04.xx, D22-23.x, D48.x). Among cancer patients, those having regional or distant metastases at diagnosis were identified by the following codes: ICD-9196-198.x; ICD-10 C77-79.x. These codes only specified the location of the metastatic tumor, not that of the primary tumor. Patients without cancer history were selected randomly and included patients with basal cell carcinoma and squamous cell carcinoma of the skin (ICD-9173.x; ICD-10 C43.x).

The electronic databases also contained vital status and demographic information such as sex, age, race, insurance type, and smoking history. The age at the time of the first cancer diagnosis or at time of visit (non-cancer patients) was used in order to categorize patients into age groups for analyses (18–34, 35–49, 50–64, 65–74, and > 75 years old). Clinical conditions existing at the time of patient’s first cancer diagnosis were identified, including autoimmune disorders. Current medication regimen or clinical activity of autoimmune disorder was unavailable. This investigation was approved by the University of Connecticut Health Institutional Review Board.

### Outcome variable (autoimmune disorders)

Using ICD codes, we ascertained presence of autoimmune disorders identified at or before the cancer diagnosis. A list of 317 ICD codes corresponding to 98 autoimmune conditions were queried in the database (eTable 1). Nonspecific codes that may refer broadly to infectious, allergic, and multiple other causes were excluded, such as “acute pancreatitis, unspecified” (577.0, K85.0). Autoimmune conditions developed after the first recorded date of cancer diagnosis were excluded.

### Statistical analyses

The chi-square test was performed to compare differences in autoimmune prevalence in the four patient groups. Multiple logistic regression was performed to characterize the relationship between autoimmune status and age groups. Relevant demographic and clinical factors were analyzed as potential confounders, which included sex, race, smoking history, cancer type, and presence of metastases. A second regression analysis was performed that considered insurance, as a proxy for age and income, instead of age group; insurance and age group were studied in separate models due to the inherent correlation between age and Medicare status. Odds ratios were calculated with 95% confidence intervals using the coefficient of the regression’s best fit line. Statistical significance was set at *p* ≤ 0.05; *p*-values were modified for multiple comparisons using the Bonferroni adjustment. Analyses were performed in RStudio.

## Results

We studied 291,333 patients aged 18–106 years old in order to ascertain potential age-related differences in autoimmunity among patients with and without cancer histories (Table 1). In all groups, there was a majority of female patients. The median age in all study groups was between 62 and 64 years old. The majority of patients were first diagnosed between 2000 and 2010 and had Medicare coverage.

**Table 1:** Characteristics of Patients by Cancer Status

	Melanoma n (%)^**a**^	Melanoma and Non-Cutaneous Cancer n (%)	Non-Cutaneous Cancer n (%)	No History of Cancer^**b**^ n (%)
**Total**	6543	2046	27,630	255,114
**Sex**				
Male	3345 (51.1)	1039 (50.8)	11,309 (40.9)	108,568 (42.6)
Female	3198 (48.9)	1007 (49.2)	13,736 (59.0)	146,536 (57.4)
Undetermined	n/a	n/a	1 (< 1)	10 (< 1)
**Age at Presentation, y**				
Median	62	64	62	62
18–34	393 (6.0)	67 (3.3)	1552 (5.6)	24,415 (9.6)
35–49	1182 (18.1)	333 (16.3)	4083 (14.8)	35,915 (14.1)
50–64	2033 (31.1)	670 (32.7)	9529 (34.5)	83,749 (32.8)
65–74	1376 (21.0)	476 (23.3)	5981 (21.6)	43,892 (17.2)
75+	1559 (23.8)	500 (24.4)	6485 (23.5)	67,143 (26.3)
**Race/ethnicity**				
White, non- Hispanic	5269 (80.5)	1927 (94.2)	23,097 (83.6)	184,554 (72.3)
Black, non-Hispanic	16 (< 1)	33 (1.6)	2491 (9.0)	17,818 (6.9)
Hispanic	8 (< 1)	2 (< 1)	452 (1.6)	8422 (3.3)
Asian & Pacific Islander	8 (< 1)	3 (< 1)	291 (1.1)	3063 (1.2)
Other^c^	1 (< 1)	n/a	30 (< 1)	304 (< 1)
Unknown	1241 (19.0)	81 (4.0)	1269 (4.6)	40,953 (16.1)
**Year**				
2000–2010	4163 (63.6)	1285 (62.8)	18,303 (66.2)	152,542 (59.8)
2011–2018	2380 (36.4)	761 (37.2)	9327 (33.8)	102,572 (40.2)
**Health Insurance**				
Medicare	3370 (51.5)	1243 (60.8)	14,836 (53.7)	113,240 (44.4)
Medicaid	135 (2.1)	75 (3.7)	2662 (9.6)	25,418 (10.0)
Private	2964 (45.3)	706 (34.5)	9764 (35.3)	108,291 (42.4)
Self-pay	74 (1.1)	22 (1.1)	367 (1.3)	8128 (3.2)
Other^d^	n/a	n/a	1 (< 1)	37 (< 1)

^a^Percentages refer to total size of study group

^a^Includes patients with basal and squamous cell skin cancer

^c^Native American/Alaskan Native and Multiracial

^d^Described as ‘other,’ ‘refunds,’ ‘contract-other,’ or ‘contract revenue’

### Prevalence of autoimmune disorders

The five most prevalent disorders among all patients were: Lichen planus (1.5%), type 1 diabetes (1.2%), rheumatoid arthritis (0.9%), systemic lupus erythematosus (0.7%), and scleroderma (0.5%; eTable 2). In all study groups, women had a higher prevalence of autoimmunity.

**Fig. 1:**
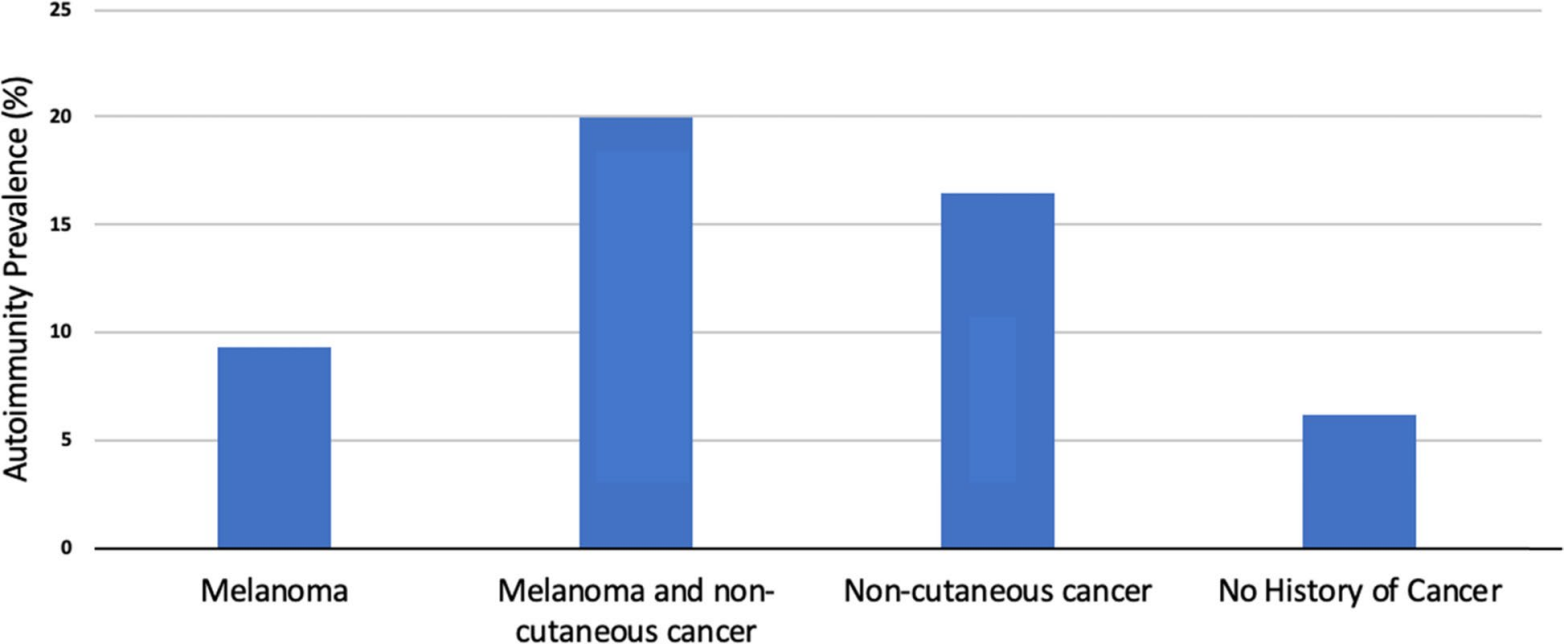
Autoimmunity prevalence by cancer group. The “No History of Cancer” group includes patients with basal and squamous cancers

Autoimmune prevalence was found to vary by study group. In the absence or presence of melanoma, a history of non-cutaneous cancers was associated with a higher prevalence of autoimmunity (Fig. 1); among those with both melanoma and non-skin cancers, the prevalence of autoimmunity was 20.0% (95% CI 18.3–21.7), and among those with non-cutaneous cancers alone, the prevalence was 16.5% (95% CI 16.1–16.9). Both rates were approximately triple that among patients without cancer history (6.2, 95% CI 6.1–6.3, *p* < 0.001). Of note, melanoma patients without a history of non-cutaneous cancer had the lowest prevalence of autoimmunity across the cancer groups (9.3, 95% CI 8.6–10.0). Further analyses demonstrated increased prevalence of autoimmunity among patients with benign vs malignant tumors, control patients with non-melanoma skin cancers vs those without, and melanoma patients treated before vs after 2011 (eTable 3).

### Patients with metastatic cancer at diagnosis

A total of 2285 patients with evidence of metastatic disease were studied (eTable 4). Patients with metastatic non-cutaneous cancer were considerably older than their group in aggregate (median 69 years vs 62), while metastatic melanoma patients with and without non-cutaneous cancers had comparable median ages to their group in aggregate (65 vs 64 and 60 vs 62, respectively). We show evidence of variability in metastasis prevalence by cancer group. Only 1.8% of patients with primary melanoma alone had metastases, compared to 8.3% of non-cutaneous cancer patients and 24.0% of patients with both melanoma and non-cutaneous cancers (eTable 3). As seen with all other analyses, women with metastases had a higher prevalence of autoimmune conditions compared to men diagnosed with metastatic disease (eTable 5).

Autoimmune prevalence among those with metastases appeared similar to that of their corresponding study group without metastases (Table 2), with the exception of the study group with both melanoma and non-cutaneous cancers: those with metastases had a higher autoimmune disease prevalence of 24.0% (95% CI 20.1–27.9) compared to 19.1% (95% CI 17.1–21.1) in patients without metastases (*p* = 0.020).

**Table 2:** Autoimmune Prevalence by Metastatic Cancer

	Melanoma (***n*** = 116)	Melanoma and Non-Cutaneous Cancer (***n*** = 471)	Non-Cutaneous Cancer^**b**^ (***n*** = 1698)
**Autoimmune Prevalence (%)**^**a**^	12 (10.3)	113 (24.0)	281 (16.5)
**Age at presentation, y**			
18–34	1 (14.3)	3 (25.0)	6 (22.2)
35–49	2 (9.1)	20 (27.0)	10 (8.9)
50–64	3 (7.1)	29 (32.2)	64 (13.1)
65–74	2 (10.5)	23 (20.1)	116 (21.4)
75+	3 (12.0)	38 (31.4)	87 (16.2)

^a^Percentages refer to respective size of subgroup among metastatic cancer

^b^Includes patients with basal and squamous cell skin cancer

### Risk factors associated with autoimmunity

A multiple logistic regression was conducted in order to identify independent factors associated with the presence of autoimmune conditions among all patients studied. Age groups greater than age 34 had increased odds of autoimmunity relative to the 18–34 age group (Table 3). Further, the 75 and older age group, compared to the 18–34 age group, was correlated with the highest odds of autoimmunity out of all age groups (Adjusted OR, 1.78, 95% CI 1.67–1.89; *p* < 0.001), which was found to be higher than females versus males (Adjusted OR, 1.53, 95% CI 1.48–1.57) or a history of melanoma versus no cancer (Adjusted OR, 1.59, 95% CI 1.46–1.73). The two greatest factors associated with autoimmunity overall were a history of non-cutaneous cancer with and without melanoma (Adjusted OR, 3.22, 95% CI 2.87–3.61; 2.46, 95% CI 2.37–2.56, respectively; *p* < 0.001). A calculation of the interaction contrast for this model determined that non-cutaneous cancer has an additive effect on the relationship between melanoma history and autoimmunity (IC = 0.31 ± 0.11, eFig. 1). There is additionally a multiplicative effect of non-cutaneous cancer on the relationship between melanoma and autoimmunity as evidenced by the distinct 95% confidence intervals. Non-Hispanic blacks had a significantly higher odds of autoimmunity relative to non-Hispanic white race (Adjusted OR 1.22, 95% CI 1.16–1.29, *p* < 0.001). Among all races, Native Americans had the highest odds of autoimmunity, but this effect was not significant after performing a *p*-value adjustment for multiple comparisons (Adjusted OR, 1.72, 95% CI 1.07–2.78, *p* = 0.4642).

**Table 3:** Multivariable Logistic Regression Analysis of Risk Factors for Autoimmunity^a^

Predictor	Unadjusted		Adjusted	
	OR (95% CI)	***p***-value^**b**^	OR (95% CI)	***p***-value^**b**^
**Age**				
18–34	1.00		1.00	
35–49	1.43 (1.34–1.52)	< 0.001	1.30 (1.22–1.39)	< 0.001
50–64	1.27 (1.20–1.35)	< 0.001	1.38 (1.30–1.46)	< 0.001
65–74	1.27 (1.19–1.35)	< 0.001	1.36 (1.27–1.45)	< 0.001
75+	1.63 (1.48–1.57)	< 0.001	1.78 (1.67–1.89)	< 0.001
**Sex**^**c**^				
Male	1.00		1.00	
Female	1.53 (1.48–1.57)	< 0.001	1.53 (1.48–1.57)	< 0.001
**Race**^**c**^				
White, non-Hispanic	1.00		1.00	
Black, non-Hispanic	1.16 (1.10–1.22)	< 0.001	1.22 (1.16–1.29)	< 0.001
Hispanic	0.80 (0.74–0.88)	< 0.001	1.04 (0.95–1.14)	1.00
Asian/Pacific-Islander	1.04 (0.91–1.18)	1.00	1.13 (1.00–1.29)	0.9780
Native American/Alaskan Native	1.68 (1.05–2.68)	0.529	1.72 (1.07–2.78)	0.4642
Multiracial	0.76 (0.40–1.44)	1.00	1.02 (0.54–1.95)	1.000
**Smoker Status**^**c**^				
Never Smoker	1.00		1.00	
Current or Past Smoker	1.07 (1.03–1.11)	0.015	1.08 (1.03–1.12)	0.005
**Cancer History**				
No History of Cancer^d^	1.00		1.00	
Melanoma Alone	1.55 (1.43–1.69)	< 0.001	1.59 (1.46–1.73)	< 0.001
Melanoma and Non- Cutaneous Cancer	3.81 (3.41–4.25)	< 0.001	3.22 (2.87–3.61)	< 0.001
Non-Cutaneous Cancer	3.00 (2.89–3.10)	< 0.001	2.46 (2.37–2.56)	< 0.001
**Metastases at Diagnosis**				
Metastases Absent	1.00			
Metastases Present	2.76 (2.48–3.08)	< 0.001	1.05 (0.94–1.18)	1.000

^a^Global *p*-value < 0.001; AIC: 146,098

^b^Individual *p*-values are Bonferroni corrected using the total number of comparisons (*n* = 18)

^c^Data for patients with unknown status not shown but included in model

^d^Includes patients with basal and squamous cell skin cancer

A second multiple regression model that considered insurance type instead of age group showed that Medicare patients had the highest risk of autoimmunity among all insurance types relative to private insurance holders (OR, 1.40, 95% CI 1.36–1.45; *p* < 0.001; eTable 6). Medicaid status did not significantly affect odds of autoimmunity (OR, 1.02, 95% CI 0.97–1.08; *p* = 0.383). Both multiple regression models analyzing age and insurance type had similar Akaike information criterion (AIC) values (146,098; 145,682, respectively) and had significant correlation for the entire model (*p* < 0.001 for both). All univariate comparisons (unadjusted) were consistent with their multivariate counterparts.

## Discussion

### Prevalence of autoimmunity by age

Our results suggest that prevalence of autoimmunity generally increases by age, particularly age > 75 years old, consistent with prior work and hypotheses in gerontology [13, 14]. This prevalence appears to vary by tumor type in that patients with a history of non-cutaneous cancers compared to those without a history of non-cutaneous cancers had significantly higher prevalence of autoimmunity. History of having had both melanoma and non-cutaneous cancer exhibited the highest association with autoimmunity compared to the other three study groups (melanoma, non-cutaneous cancer, or no cancer history) .

Our findings appear to be supported by the current understanding of aging physiology. Increased tumor burden, either in metastatic disease or advanced age, are likely associated with pro-inflammatory cytokines that contribute to autoimmunity [15, 16]. Aging is shown to promote the ability of CD4+ T cells to generate an IL-17 response that promote autoimmunity in humans [17]. Tumor neoantigens have been linked to immune responses that have capacity to cross react with host tissues. Scleroderma is a well-studied example wherein patients diagnosed with lung cancer can develop interstitial lung disease through unique antibodies to tumor epitopes [18]. Similarly, there are associations between occult malignancy and dermatomyositis, polymyositis, rheumatoid arthritis, and systemic lupus erythematosus [19, 20]. It has been posited that autoimmunity may be reactive to an occult malignancy in the absence of an infectious state [21]. This theory may provide some rationalization as to why rates of autoimmunity were relatively high in patients with both benign and malignant tumors in our investigation. Additionally, autoimmune disorders can promote an inflammatory state that leads to cancer, particularly in lymphomas.

While the connection between aging and cancer is well-established, our study is the first large-scale observational study using real-world data, to our knowledge, reporting associations with autoimmunity. Given the link between autoimmunity and moderate to severe adverse events in ICI therapy, current NCCN Guidelines state that patients with neurologic and life-threatening autoimmune disease are recommended against receiving therapy [22]. Recommendations for senior patients and a full range of autoimmune disorders, however, are not included in recommendations. Further, the marked increase in autoimmunity odds observed at age 75 suggests associated changes in the immune system are seen in this population. This finding also suggest that age of 75 years also represent a critical point for collection of pertinent patient history prior to treatment with ICIs.

Future epidemiologic studies of prevalence of autoimmunity across the life span are suggested as there is a dearth of evidence to date in cancer patients. Given this lack of knowledge, it is difficult to place our prevalence data in context. For example, our overall autoimmunity prevalence of 7.3%, however, is somewhat consistent NIH 2005 estimate in the U.S. (5–8%), but higher than the worldwide estimate from Hayter’s meta-analysis (4.6%) [23, 24]. Our dataset notably looked at 98 conditions instead of a list of 81 conditions reported previously by Hayter. As with all EHR studies, such as ours, there are potential diagnostic inaccuracies that may impact our results. For example, urticaria secondary to an allergen can be incorrectly coded as “idiopathic” (ICD-9: 708.1, ICD-10: L50.1) when the condition should have been coded as “allergic urticaria” (ICD-9: 708.0, ICD-10: L50.0).

### Prevalence of autoimmunity in cancer patients

Our data demonstrate that cancer patients have a higher prevalence of autoimmunity compared to the non-cancer patient population. Cancer patients have an increased risk of subsequent autoimmunity, such as in paraneoplastic syndromes, and patients with certain autoimmune conditions have an increased risk of cancer [25, 26]. As autoimmune diagnoses were largely coded in the database at the time of cancer diagnosis, it remains uncertain which diagnosis came first. Thus, our work cannot support either directional arrow. Interestingly, while the autoimmune disease prevalence among melanoma patients (9.3%) was significantly higher than that of the non-cancer population (6.2%), the prevalence was significantly lower than those with a history of non-cutaneous cancer, with or without melanoma. The data are roughly consistent with limited past epidemiological studies on cancer patients. In those with lung and renal cancer, the prevalence of autoimmunity, using a shortened list of about 40 autoimmune conditions, is estimated to be 25% [27, 28]. In one study that examined melanoma patients and included 147 different autoimmune diagnoses, the prevalence of autoimmunity was found to be 20.5%, which is close to our rate of melanoma patients with non-cutaneous cancer [29].

Our study showed a significant effect of metastases in patients with both melanoma and non-cutaneous cancers. These patients had a 26% metastases-associated increase in autoimmune prevalence. Our results are similar to a study that that used a looser case definition for autoimmunity and found a 43% relative increase in autoimmune disease prevalence among melanoma patients with metastases compared to those without [29]. The disparate findings suggest that the specificity of autoimmune case definitions may impact results.

### Sex and race

We found an odds ratio for autoimmunity of 1.53 for female sex vs male sex, which is slightly less than the relative risk of 2.4 calculated by Hayter et al. [26] Our data also show that non-Hispanic black and Native American race are associated with autoimmunity, which is consistent with a recent large national database study that found an association with these groups and multiracial individuals [30]. We did not find a significant association between Asian-American or multiracial race and autoimmunity, but their results were limited somewhat by small sampling. While the influence of sex and race on autoimmunity is incompletely understood, it is thought to be a combination of genetic and environmental factors [30].

### Limitations

This study is limited by incomplete clinical information in the medical record as well as inherent generalizability concerns from a single-institutional report. For example, severity, control, and activity of autoimmune conditions were not available consistently. Similarly, prognostic factors such as frailty, Charlson Comorbidity Index, ECOG score, and specific cancer stage or grade were not included in analyses. Additionally, the database does not specify which cancer site is linked to ICD codes of metastases, so it is uncertain whether melanoma metastases impact autoimmunity in the combined melanoma and non-cutaneous group. Selection bias might be a concern given that our sample is not population-based. Regarding the link between age and autoimmunity, for example, young patients are less likely to seek routine medical attention in a hospital-based clinic and may receive fewer diagnoses of autoimmunity as a result. However, to illustrate, aged 18–34 years old represented 9.6% of the study group without cancer compared to 3.3–6.0% in the cancer study groups.

## Conclusions

Our findings highlight how non-cutaneous cancer history, non-Hispanic black race, Native American race, and age > 75 are strongly associated with underlying autoimmunity. While we did not study irAEs, the significantly increased prevalence of autoimmunity in these clinical sub-groups, particularly the older patient, suggests a higher risk for adverse events during ICI treatment as pre-existing autoimmunity has been shown to increase this likelihood [3, 4]. Structured history-taking and closer monitoring in patients > 75 undergoing ICI therapy, therefore, may be recommended as they are increasingly becoming eligible for this therapy, and autoimmune manifestations of underlying disease may be atypical, undiagnosed, or occult in this population [13]. Future prospective studies are needed to determine causal links between autoimmunity, irAE development, and survivorship.

## Supplementary Information

**Additional file 1 **: **eFigure 1**. Risk difference for autoimmunity between patients with melanoma alone versus those with melanoma and non-cutaneous cancers. An additive interaction was identified using the odds of the fitted multiple regression model for autoimmune status that included cancer history, age, sex, race, smoking history, and presence of metastases. The interaction contrast was found to be 0.29 ± 0.12. Bars indicate 95% confidence interval for risk differences. **eTable 1**. Autoimmune Conditions Queried in Database. **eTable 2**. Most Common Autoimmune Conditions Studied by Cancer Status. **eTable 3**. Comparison of Autoimmune Prevalence Among Select Groups^a^. **eTable 4**. Summary Statistics for Patients With Metastases. **eTable 5**. Autoimmune Prevalence by Metastatic Cancer Type with Additional Parameters. **eTable 6**. Multivariate Logistic Regression Analysis of Factors Predicting Autoimmunity with Insurance Type^a^.

## Data Availability

The datasets generated and analyzed during the current study are not publicly available due to its inclusion of health information, but are available from the corresponding author on reasonable request.

## Abbreviations

AIC: Akaike information criterion
ICD: International Classification of Disease
ICI: Immune checkpoint inhibitor
irAE: Immune-related adverse events
