# Supplementary material for: Association of advanced age and cancer history with autoimmune disease in melanoma patients: a cross-sectional study
Source: BMC Cancer. 2021 Dec 6;21:1300. doi: 10.1186/s12885-021-09001-1 (PMC8647353; doi:10.1186/s12885-021-09001-1)
Supplement: Supplementary file 1 — Additional file 1 : eFigure 1. Risk difference for autoimmunity between patients with melanoma alone versus those with melanoma and non-cutaneous cancers. An additive interaction was identified using the odds of the fitted multiple regression model for autoimmune status that included cancer history, age, sex, race, smoking history, and presence of metastases. The interaction contrast was found to be 0.29 ± 0.12. Bars indicate 95% confidence interval for risk differences. eTable 1. Autoimmune Conditions Queried in Database. eTable 2. Most Common Autoimmune Conditions Studied by Cancer Status. eTable 3. Comparison of Autoimmune Prevalence Among Select Groupsa. eTable 4. Summary Statistics for Patients With Metastases. eTable 5. Autoimmune Prevalence by Metastatic Cancer Type with Additional Parameters. eTable 6. Multivariate Logistic Regression Analysis of Factors Predicting Autoimmunity with Insurance Typea. [file 12885_2021_9001_MOESM1_ESM.docx]

**
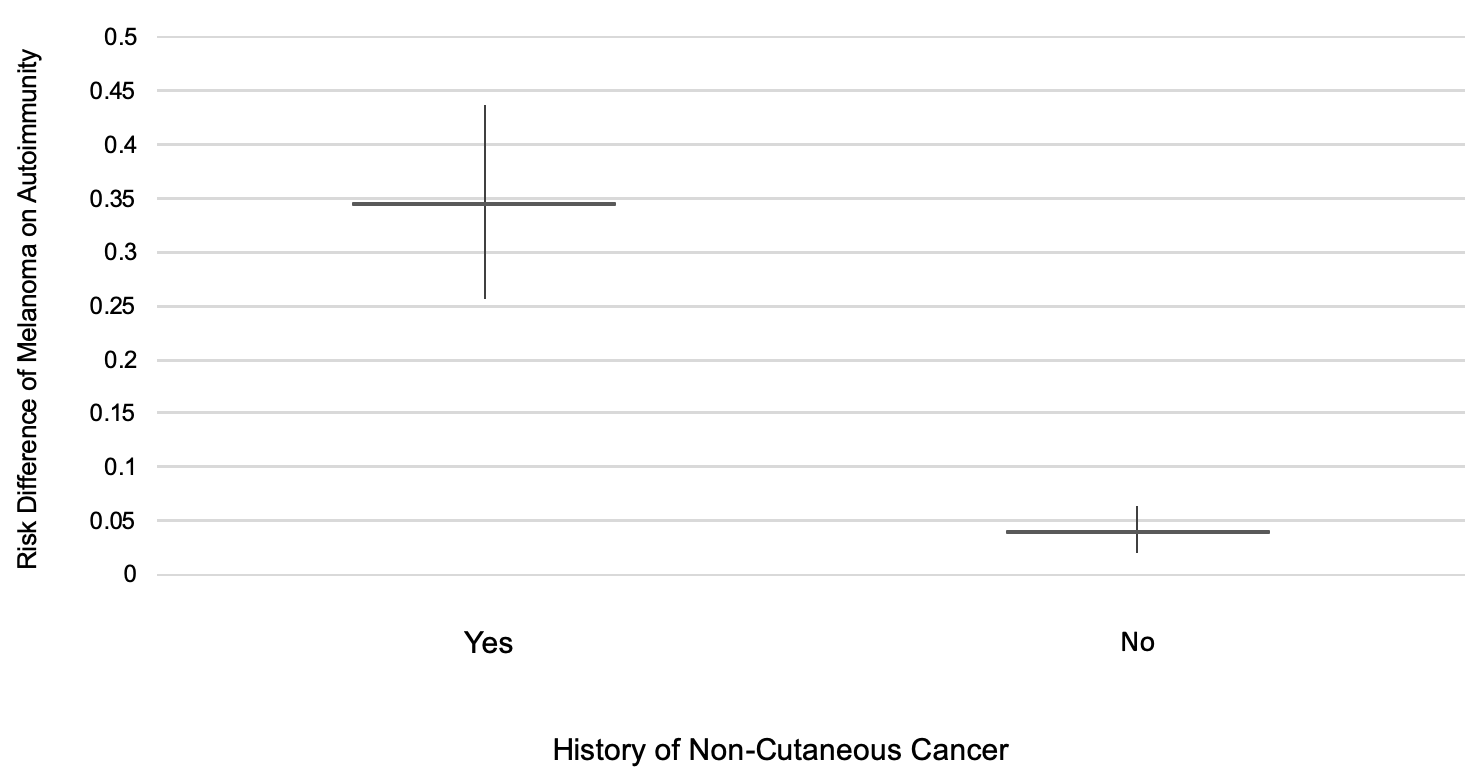
**

**eFigure 1.** Risk difference for autoimmunity between patients with melanoma alone versus those with melanoma and non-cutaneous cancers. An additive interaction was identified using the odds of the fitted multiple regression model for autoimmune status that included cancer history, age, sex, race, smoking history, and presence of metastases. The interaction contrast was found to be 0.29 ± 0.12. Bars indicate 95% confidence interval for risk differences.

**eTable 1. Autoimmune Conditions Queried in Database**

| **Condition** | **ICD-9 Code(s)** | **ICD-10 Code(s)** | **Exclusions** |
| --- | --- | --- | --- |
| Autoimmune Myocarditis | 391.2 | I01.2 | 421.0, I33.0 |
| Postmyocardial infarction syndrome | 411 | I24.1 | 429.4, I97.0 |
| Anti-Glomerular Basement Membrane nephritis | 446.21 | M31.0 |  |
| Interstitial cystitis | 595.1 | N30.10,N30.11 |  |
| Autoimmune hepatitis | 571.42 | K75.4 |  |
| Primary biliary cholangitis(PBC) | 571.6 | K74.3 |  |
| Primary sclerosing cholangitis | n/a | K83.01 |  |
| Alopecia Areata | 704.01 | L63.9,L63.2,L63.8 |  |
| Autoimmune Angioedema | 995.1 | T78.3XXA |  |
| Bullous pemphigoid | 694.5 | L12.9,L12.0,L12.8 |  |
| Cicatricial pemphigoid | 694.61 | L12.1 |  |
| Dermatitis herpetiformis | 694 | L13.0 |  |
| Discoid lupus erythematosus | 695.4 | L93.0 |  |
| Epidermolysis bullosa acquisita | 694.8 | L14,L13.8 |  |
| Erythema nodosum | 695.2 | L52 |  |
| Hidradenitis suppurativa | 705.83 | L73.2 |  |
| Lichen planus | 697 | L43.0,L43.1,L43.2,L43.3,L43.9,L43.8,L66.1 |  |
| Lichen sclerosus | 701 | L94.1,L90.0,L94.0,L94.3 |  |
| Pemphigus vulgaris | 694.4 | L10.3,L10.5,L10.81,L10.89,L10.0,L10.1,L10.2,L10.4,L10.9 |  |
| Pityriasis lichenoides et varioliformis acuta | 696.2 | L41.0 ,L41.1 ,L41.8,L41.3,L41.4,L41.5,L41.9,L94.5, |  |
| Psoriasis | 696 | L40.50,L40.51,L40.52,L40.53,L40.54,L40.59 , |  |
| Systemic scleroderma | 710.1 | M34.2,M34.81,M34.82,M34.83,M34.89,M34.0,M34.1,M34.9, |  |
| Vitiligo | 709.01 | L80 |  |
| Addison's disease | n/a | E27.1 ,E27.2 |  |
| Autoimmune polyendocrine syndrome (APS) type 1 | 258.1 | E31.0 |  |
| Autoimmune polyendocrine syndrome (APS) type 2 | 258.1 |  |  |
| Autoimmune polyendocrine syndrome (APS) type 3 | 258.1 | E31.0 |  |
| Diabetes mellitus type 1 | 250.01,250.03 | E10.9,E10.65 |  |
| Autoimmune thyroiditis | 245.8, 245.2 | E06.2,E06.5,E06.3,E06.1 | 245.4 |
| Graves' disease | 242.00,242.01 | E05.00,E05.01 |  |
| Autoimmune Oophoritis | 614.2 | N70.91,N70.92,N70.93 |  |
| Autoimmune orchitis | 604 | N45.4 | 604.0, K12.1x, N45.4 |
| Sjogren's syndrome | 710.2 | M35.02,M35.03,M35.04,M35.09,M35.00,M35.01 |  |
| Coeliac disease | 579 | K90.0 |  |
| Crohn's disease | 555 | K50.011,K50.012,K50.013,K50.014,K50.018,K50.00 , |  |
| Microscopic colitis | n/a | K52.831,K52.832,K52.838,K52.839,K52.3,K52.89 | 558.9, K52.9 |
| Ulcerative colitis | 556.0,556.1 | K51.80 |  |
| Antiphospholipid syndrome(APS, APLS) | 289.81 | D68.52,D68.59,D68.61,D68.62 | D68.51, K52.892, D58.52 |
| Aplastic anemia | 284.9 | D61.9 |  |
| Autoimmune hemolytic anemia | 283 | D59.0,D59.1 |  |
| Autoimmune lymphoproliferative syndrome | 279.41 | D89.82 |  |
| Autoimmune thrombocytopenic purpura | 287.31 | D69.3 | 287.0, D69.0 |
| Essential mixed cryoglobulinemia | 273.2 | D89.1 |  |
| Evans syndrome | 287.32 | D69.41 |  |
| Pernicious anemia | 281 | D51.0 |  |
| Pure red cell aplasia | 284.81 | D60.0,1D60.0,D60.8,D60.9 |  |
| Adult-onset Still's disease | 714.2 | M05.20,M05.211,M05.212,M05.219,M05.221,M05.30 ,M05.60 ,M06.1 |  |
| Ankylosing spondylitis | 720 | M08.1,M45.0,M45.1,M45.2,M45.3,M45.9 |  |
| CREST syndrome | 710.1 | M34.1 |  |
| Drug-induced lupus | 710 | M32.0 |  |
| Enthesitis-related arthritis | n/a | M08.80 |  |
| Eosinophilic fasciitis | n/a | M35.4 | 728.89 |
| Felty syndrome | 714.1 | M05.011,M05.012,M05.019,M05.021,M05.022,M05.00 |  |
| Juvenile arthritis | 714.3 | n/a |  |
| Lyme disease (chronic) |  | A69.20 |  |
| Mixed connective tissue disease (MCTD) | 710.8 | M04.2,M04.8,M35.1,M35.8,M35.5 |  |
| Palindromic rheumatism | 719.3 | M12.30 |  |
| Psoriatic arthritis | 696 | L40.50,L40.51,L40.52,L40.53,L40.54,L40.59 |  |
| Reactive arthritis | 99.3 | M02.311,M02.312,M02.319,M02.321,M02.322,M02.30 |  |
| Rheumatic fever without heart involvement | 390,391.9, | I00 ,I01.9 |  |
| Rheumatic fever wit heart involvement | 390.1,391.2,391.9 | I01.1,I01.2,I01.9 |  |
| Rheumatoid arthritis | 714 | M05.20,M05.211,M05.212,M05.219,M05.221,M05.30 ,M05.60 ,M06.1 |  |
| Sarcoidosis | 135 | D86.0,D86.1,D86.2,D86.3,D86.81,D86.9 |  |
| Systemic Lupus Erythematosus (SLE) | 695.4 | L93.1,L93.2 |  |
| Dermatomyositis | 710.3 | M33.00,M33.01,M33.02,M33.09,M33.10,M33.03,M33.13,M33.90,M33.93 |  |
| Inclusion body myositis | 359.71 | G72.41 |  |
| Myositis | n/a | M60.80,M60.811,M60.812,M60.819,M60.821,M60.9 | 729.1, M79.1 |
| Myasthenia gravis | 358.00,358.01 | G70.00,G70.01 |  |
| Polymyositis | 710.4 | M33.21,M33.22,M33.29,M33.20 |  |
| Balo concentric sclerosis | 341.1 | G37.5 |  |
| Chronic inflammatory demyelinating polyneuropathy | 357.81 | G61.81 |  |
| Guillain–Barré syndrome | 357 | G61.0 |  |
| Lambert-Eaton myasthenic syndrome | 358.1 | G73.3 |  |
| Multiple sclerosis, pattern II | 340 | G37.0,G35 , |  |
| Stiff person syndrome | 333.91 | G25.82 |  |
| Sydenham chorea | 392 | I02.0 |  |
| Transverse myelitis | 341.2 | G37.3 |  |
| Autoimmune uveitis | 364.04 |  |  |
| Cogan syndrome | n/a | H20.041,H20.042,H20.043,H20.049 | 370.52, H16.321 |
| Ligneous conjunctivitis | 372.39 | H10.511,H10.512,H10.513,H10.519 |  |
| Mooren's ulcer | 370.07 | H16.051,H16.052,H16.053,H16.059 |  |
| Neuromyelitis optica | 341 | G36.0 |  |
| Scleritis | 379 | H15.001,H15.002,H15.003,H15.101,H15.102,H15.009,H15.109 |  |
| Sympathetic ophthalmia | 360.11 | H44.131,H44.132,H44.133,H44.139 |  |
| Ménière's disease | 386 | H81.01,H81.02,H81.03,H81.09 |  |
| Behçet's disease | 136.1 | M35.2 |  |
| Eosinophilic granulomatosis with polyangiitis (EGPA) | 446.4 | ,M30.1 |  |
| Giant cell arteritis | 446.5 | M31.6,M31.5 |  |
| Granulomatosis with polyangiitis (GPA) | 446.4 | M31.30,M31.31 |  |
| Kawasaki's disease | 446.1 | M30.3 |  |
| Lupus vasculitis | 695.4 | L93.2 |  |
| Microscopic polyangiitis(MPA) | 446 | M31.7 |  |
| Polyarteritis nodosa (PAN) | 446 | M30.0 |  |
| Polymyalgia rheumatica | 725 | M35.3 |  |
| Non-allergic urticaria | 708.9 | L50.9 | 708.0, L50.0 |
| Vasculitis | 447.6 | I77.6 |  |
| Paroxysmal nocturnal hemoglobinuria (PNH) | 283.2 | D59.5 |  |
| Vogt-Koyanagi-Harada Disease | 364.24 | H20.823, H20.821 |  |
| Unspecified autoimmunity | 279.49 | D89.89 |  |

**eTable 2. Most Common Autoimmune Conditions Studied by Cancer Status.**

|  | **Melanoma (n=692)** | **Melanoma and Non-Cutaneous Cancer (n=639)** | **Non-Cutaneous Cancer**  **(n=6,973)** | **No History of Cancer^a^**  **(n=22,866)** |
| --- | --- | --- | --- | --- |
| **1.** | Lichen Planus  (352) | Lichen Planus (122) | Type 1 Diabetes (960) | Lichen Planus (3,312) |
| **2.** | Parapsoriasis  (50) | Type 1 Diabetes (59) | Rheumatoid Arthritis (551) | Type 1 Diabetes (2,572) |
| **3.** | Rheumatoid Arthritis (25) | Rheumatoid Arthritis (39) | Lichen Planus (483) | Rheumatoid Arthritis (2,105) |
| **4.** | Type 1 Diabetes (21) | Scleroderma (36) | Systemic Lupus Erythematosus (328) | Systemic Lupus Erythematosus (1,595) |
| **5.** | Non-allergic Urticaria (14) | Systemic Lupus Erythematosus (26) | Graves’ Disease (324) | Scleroderma (1,107) |
| **6.** | Pemphigus Vulgaris, Bullous Pemphigoid, & Alopecia Areata (13) | Parapsoriasis (25) | Hashimoto’s Thyroiditis (261) | Parapsoriasis (1,087) |
| **7.** | Localized Scleroderma & Systemic Lupus Erythematosus (12) | Non-allergic Urticaria (21) | Non-allergic Urticaria (260) | Sjogren’s Syndrome (1,024) |
| **8.** | Arteritis or Vasculitis, unspecified (10) | Pernicious Anemia & Graves’ Disease (18) | Scleroderma (248) | Non-allergic urticaria (666) |
| **9.** | Scleroderma & Psoriatic Arthritis (9) | Sjogren’s Syndrome (17) | Sjogren’s Syndrome (244) | Bullous pemphigoid  (603) |
| **10.** | Celiac Disease (8) | Hashimoto’s Thyroiditis (16) | Pernicious Anemia (239) | Alopecia areata (530) |

^a^Includes patients with basal and squamous cell skin cancers.

**eTable 3. Comparison of Autoimmune Prevalence Among Select Groups^a^**

| **Comparison** | **Sample size, n, and autoimmune prevalence (%) of group 1** | **Sample size, n, and autoimmune prevalence (%, 95% CI) of group 2** | **p-value** |
| --- | --- | --- | --- |
| **Benign vs malignant tumors^b^** | 14,033 (17.5, 16.9-18.1) | 12,130 (15.8, 95% CI 15.2-16.4) | < 0.001 |
| **Patients with no cancer history vs those with non-melanoma skin cancers only** | 203,766 (5.8, 95% CI 5.7-5.9) | 51,348 (7.6, 95% CI 7.4-7.8) | < 0.001 |

^a^Results for two groups are organized respective to order in “Comparison” column

^b^Excluding melanoma and skin cancers

**eTable 4. Summary Statistics for Patients With Metastases**

|  | **Melanoma n (%)^a^** | **Melanoma and Non-Cutaneous Cancer n (%)^a^** | **Non-Cutaneous Cancer**  **n (%)^a^** |
| --- | --- | --- | --- |
| **Total** | 116 | 471 | 1,698 |
| **Sex** |  |  |  |
| Male | 64 (55.2) | 258 (54.8) | 803 (47.3) |
| Female | 52 (44.8) | 213 (45.2) | 895 (52.7) |
| Undetermined | n/a | n/a | n/a |
| **Age** |  |  |  |
| Median | 60 | 65 | 69 |
| 18-34 | 7 (6.0) | 14 (3.0) | 27 (1.6) |
| 35-49 | 22 (19.0) | 74 (15.7) | 112 (6.6) |
| 50-64 | 43 (37.1) | 153 (32.5) | 490 (28.9) |
| 65-74 | 19 (16.4) | 109 (23.1) | 531 (31.3) |
| 75+ | 25 (21.6) | 121 (25.7) | 538 (31.7) |
| **Race/ethnicity** |  |  |  |
| White, non-Hispanic | 114 (98.3) | 448 (95.1) | 1,510 (88.9) |
| Black, non-Hispanic | n/a | 11 (2.3) | 129 (7.6) |
| Hispanic | n/a | 1 (<1) | 32 (1.9) |
| Asian & Pacific  Islander | n/a | 1 (<1) | 12 (<1) |
| Other^b^ | n/a | n/a | 1 (<1) |
| Unknown | 2 (1.7) | 10 (2.1) | 14 (<1) |

^a^Percentages refer to total size of metastasis patients by study group

^b^Native American/Alaskan Native and Multiracial

**eTable 5. Autoimmune Prevalence by Metastatic Cancer Type with Additional Parameters.**

|  | **Melanoma (n=116)** | **Melanoma and Non-Cutaneous Cancer (n=471)** | **Non-Cutaneous Cancer**  **(n=1,698)** |
| --- | --- | --- | --- |
| **Autoimmunity Prevalence (%)^a^** | 12 (10.3) | 113 (24.0) | 281 (16.5) |
| **Sex** |  |  |  |
| Male | 6 (9.4) | 54 (20.9) | 123 (15.3) |
| Female | 6 (11.5) | 59 (27.7) | 158 (17.7) |
| Undetermined | n/a | n/a | n/a |
| **Race/ethnicity** |  |  |  |
| White, non-Hispanic | 12 (10.5) | 105 (23.4) | 245 (16.2) |
| Black, non-Hispanic | n/a | 5 (45.5) | 29 (22.5) |
| Hispanic | n/a | n/a | 5 (15.6) |
| Asian & Pacific  Islander | n/a | 1 (100) | 1 (8.4) |
| Other^b^ | n/a | n/a | n/a |
| Unknown | n/a | 2 (20) | 1 (7.1) |

^a^Percentages refer to size of respective subgroup among metastasis patients.

^b^Native American/Alaskan Native and Multiracial

**eTable 6.** **Multivariate Logistic Regression Analysis of Factors Predicting Autoimmunity with Insurance Type^a^**

| **Predictor** | **Unadjusted** |  |  | **Adjusted** |  |
| --- | --- | --- | --- | --- | --- |
|  | **OR (95% CI)** | **p-value^b^** |  | **OR (95% CI)** | **p-value^b^** |
| **Insurance** |  |  |  |  |  |
| Private | 1.00 |  |  |  |  |
| Medicare | 1.48 (1.44-1.53) | < 0.001 |  | 1.40 (1.36-1.45) | < 0.001 |
| Medicaid | 1.24 (1.18-1.30) | < 0.001 |  | 1.02 (0.97-1.08) | 1.00 |
| Self-Pay | 0.59 (0.52-0.66) | < 0.001 |  | 0.63 (0.56-0.71) | < 0.001 |
| Other^c^ | 1.33 (0.41-4.33) | 1.00 |  | 1.93 (0.59-6.32) | 1.00 |
| **Sex^d^** |  |  |  |  |  |
| Male | 1.00 |  |  |  |  |
| Female | 1.53 (1.48-1.57) | < 0.001 |  | 1.52 (1.43-1.57) | < 0.001 |
| **Race^d^** |  |  |  |  |  |
| White, non-Hispanic | Ref |  |  |  |  |
| Black, non-Hispanic | 1.16 (1.10-1.22) | <0.001 |  | 1.20 (1.14-1.27) | <0.001 |
| Hispanic | 0.80 (0.74-0.88) | <0.001 |  | 1.02 (0.93-1.11) | 1.00 |
| Asian/Pacific-Islander | 1.04 (0.91-1.18) | 1.00 |  | 1.18 (1.04-1.34) | 0.217 |
| Native American/Alaskan Native | 1.68 (1.05-2.68) | 0.529 |  | 1.74 (1.08-2.81) | 0.418 |
| Multiracial | 0.76 (0.40-1.44) | 1.00 |  | 0.99 (0.52-1.82) | 1.00 |
| **Smoker Status^d^** |  |  |  |  |  |
| Never Smoker | 1.00 |  |  |  |  |
| Current or Past Smoker | 1.07 (1.03-1.11) | 0.015 |  | 1.07 (1.03-1.12) | 0.012 |
| **Cancer History** |  |  |  |  |  |
| No History of Cancer^e^ | 1.00 |  |  |  |  |
| Melanoma Alone | 1.55 (1.43-1.69) | <0.001 |  | 1.54 (1.42-1.68) | <0.001 |
| Melanoma and Non- Cutaneous Cancer | 3.81 (3.41-4.25) | <0.001 |  | 3.06 (2.72-3.43) | <0.001 |
| Non-Cutaneous Cancer | 3.00 (2.89-3.10) | <0.001 |  | 2.39 (2.30-2.48) | <0.001 |
| **Metastases at Diagnosis** |  |  |  |  |  |
| Metastases Absent | 1.00 |  |  |  |  |
| Metastases Present | 2.76 (2.48-3.08) | <0.001 |  | 1.05 (0.93-1.18) | 1.000 |

^a^Global p-value < 0.001; AIC: 145,682

^b^Individual p-values are Bonferroni corrected using the total number of comparisons (n=18)

^c^Described as ‘other,’‘refunds,’‘contract-other,’ or ‘contract revenue.’

^d^Data for patients with unknown status not shown but included in model.

^e^Includes patients with basal and squamous cell skin cancer
